# Overexpression of Annexin A1 Suppresses Pro-Inflammatory Factors in PC12 Cells Induced by 1-Methyl-4-Phenylpyridinium 

**DOI:** 10.22074/cellj.2016.4314

**Published:** 2016-05-30

**Authors:** Abbas Kiani-Esfahani, Sedigheh Kazemi Sheykhshabani, Maryam Peymani, Motahare-Sadat Hashemi, Kamran Ghaedi, Mohammad Hossein Nasr-Esfahani

**Affiliations:** 1 Department of Cellular Biotechnology, Cell Science Research Center, Royan Institute for Biotechnology, ACECR, Isfahan, Iran; 2 Cellular and Molecular Research Center, Shahrekord University of Medical Sciences, Shahrekord, Iran; 3 Department of Biology, Faculty of Basic Sciences, Shahrekord Branch, Islamic Azad University, Sahrekord, Iran; 4 Department of Biology, School of Sciences, University of Isfahan, Isfahan, Iran

**Keywords:** Annexin A1, Parkinson’s Disease, PC12 Cells, ROS

## Abstract

**Objective:**

Annexin A1 (ANXA1) is suggested to have anti-inflammatory function. However, the precise function of ANXA1 has remained unclear. In this study, we therefore examined the potency of ANXA1 in regulating reactive oxygen species (ROS) production and suppressing pro-inflammatory responses in PC12 cells induced by 1-methyl-4-phenylpyridinium (MPP+).

**Materials and Methods:**

In this experimental study, cDNA of ANXA1 was cloned and inserted to the PGL268 pEpi-FGM18F vector to produce a recombinant PGL/ANXA1 vector for transfection into the PC12 cells. ANXA1 transfected cells were then treated with MPP+. Apoptosis and the content of pro-inflammatory factors including ROS, Interlukin-6 (*IL-6*), inducible nitric oxide synthase (*iNOS*) and nuclear factor-kappa B (NF-κB) were assessed by flow-cytometry, real-time quantitative polymerase chain reaction (RT-qPCR) and western blot in ANXA1-transfected cells and the data were compared with those obtained from mock and control cells.

**Results:**

Data revealed that overexpression of ANXA1 is associated with decreased levels of ROS and expression level of *IL-6* and *iNOS* transcripts, and NF-κB protein in MPP+ treated PC12 cells.

**Conclusion:**

ANXA1 may be considered as an agent for prevention of neurodegenerative or inflammatory conditions.

## Introduction

Parkinson’s disease (PD) is characterized by death of dopaminergic neurons in *Substantia Nigra pars compacta (SNpc)*, a region of the midbrain, generation of lewy bodies and neuroinflammation (1,3). Although the exact origin of neuronal degeneration in PD has not been well understood, several hypotheses have been postulated for the etiology of PD onset including age, genetic factors, oxidative stress, mitochondrial dysfunction and environmental toxins (4,6). Neuropathological manifestations of PD are associated with neurological impairment that results from pro-inflammatory factors. These factors are the major determinants of PD pathogenesis which are induced by infectious or toxic agents (7). Neuroinflammation is associated with presence of activated microglia, reactive astrocytes and adaptive immune cells in parenchyma of central nervous system (CNS). Pro-inflammatory cytokines, and reactive oxygen and nitrogen species (ROS/RNS) are secreted by these cells which lead to the progress of PD (8). Oxidative stress and cell apoptosis play main roles in pathogenesis of PD (9). Among pro-inflammatory mediators, interlukin-1 beta (IL-1β), *IL-6*, tumor necrosis factoralpha (*TNF-α*), cyclooxygenase 2 (COX-2) and inducible nitric oxide synthase (*iNOS*) have important roles in generating inflammation (10,13). The key mediator of inflammatory responses is NF-κB, which acts as a nuclear transcription factor. Normaly, inactivated nuclear factor-kappa B (NF-κB), is bound to inhibitor of kappa B (IκB) in the cytosol. Under inflammatory conditions, phosphorylated IκB is dissociated from NF-κB. Free activated NF-κB translocates to the nucleus which induces the expression of inflammatory genes including *IL-1β, IL-6, TNF-α* and *COX-2*. Subsequently, the inflammatory condition is accompanied by the activation of mitogen-activated protein kinases (MAPKs) including extracellular signal-regulated kinase (ERK), c-Jun N-terminal kinase (JNK) and p38 (14,16). 

Annexin A1 (ANXA1) or lipocortin is a member of the annexin protein superfamily and is defined as a calcium-dependent phospholipid binding protein that plays different roles in various biological systems. Recently, it has been shown to act as a regulator not only for cellular functions such as anti-inflammatory effects, cell proliferation and apoptosis, but also for cell differentiation (17,18). Previous studies have demonstrated that ANXA1 acts as a regulator of inflammation by inhibiting inflammatory factors such as phospholipase A2 (19) and NF-κB (20). Several studies have also reported a preventive role of ANXA1 against cell death *in vitro* and *in vivo* (21,22). 

Disruption of mitochondrial complex I activity in the electron transport chain occurs in SNpc, skeletal muscle, and platelets of PD patients (23,25).1-methyl-4-phenylpyridinium (MPP+), a toxic metabolite of 1-methyl-4-phenyl-1, 2, 3, 6-tetrahydropyridine (MPTP), is a mitochondrial complex I inhibitor (26,27), which is used for induction of oxidative stress, apoptosis and inflammation, especially in dopaminergic neurons. PC12 cells have served as a convenient model cell for studying neuronal development and function. 

One of the main interests in the medical field is finding new factors for inflammation relief, especially in neurodegenerative diseases. As the potential role of ANXA1 has so far not been studied, the aim of the present study is to assess the possible inhibitory role of ANXA1 against MPP+ induced inflammation and apoptosis in PC12 cells. 

## Materials and Methods

The most reagents in this experimental study were supplied by Sigma (CA, USA) unless indicated otherwise. 

### Ethical issue

This study was approved by the Ethical Committee of Royan Institute (Project ID. 920010). 

### Cell culture and transfection of AgeI-ANXA1FLAG in PC12 cells

PC12 cells (obtained from Royan Institute for Stem Cell Biology and Technology, Iran) were placed on 0.01% poly-L-lysine-coated 6-well dish in presence of Dulbecco’s modified Eagle’s medium (DMEM, Life Technologies, CA, USA) supplemented with 10% fetal calf serum (FCS, Life Technologies), and 5% horse serum (Life Technologies) at 37˚C. PC12 cells were transfected with either pEPi FGM18F PGL-268 or pEPi FGM18F PGL-268/AgeI-ANXA1-FLAG (described in Supplementary Material Information and below) by Lipofectamine LTX reagent based on the manufacturer’s instructions (Invitrogen, USA). 

### Cell staining

Cells were cultured on glass coverslips and washed the day after with phosphate buffer saline (PBS-Life Technologies) and fixed with 4% paraformaldehyde (Sigma) in PBSfor 20 minutes at room temperature. Cells were then permeabilized with 0.2% triton X-100 (Sigma) at 37˚C for 30 minutes. Cells were washed again and incubated for 1 hour with mouse anti-tyrosine hydroxylase (TH, 1:200, Sigma). Next, cells were incubated for 1 hour with labeled rabbit anti-mouse secondary antibody (Milipore, USA). For nuclei staining, cells were incubated for 3 minutes with 10 µg/mL 4´, 6-diamidino-2-phenylindole, dihydrochloride (DAPI, Sigma) in bovine serum albumin (BSA, Sigma). After washing, coverslips were mounted on glass slides and analyzed under a fluorescent microscope (Olympus, Japan) with images acquired with an Olympus DP70 camera (Olympus, Japan). 

### Viability assay

The( 3-(4,5-dimethylthiazol-2-yl)-5-(3carboxymethoxyphenyl)-2-(4-sulfophenyl)-2Htetrazolium) (MTS) assay was performed to evaluate the number of viable cells based on mitochondrial dehydrogenase activity. Upon tetrazolium absorption into living cells, it is converted to formazan by mitochondrial dehydrogenase enzyme activity. Therefore, accumulation of formazan reflects the activity of mitochondria and is associated with cell viability. Briefly, PC12 cells (10^4^ cells/well) were plated in 96well plates (Techno Plastic Products, Switzerland) and treated with different concentrations of MPP+ for 24 hours at 37˚C. Cells were washed gently with PBS. Twenty μL of MTS (0.5 mg/mL) and 200 μL of medium were added to each well for 4 hours at 37˚C. The supernatant was removed and 150 μL of dimethyl sulfoxide (DMSO, Sigma) was added to each well. Optical density (OD) was assessed at 570 nm in an ELISA microplate reader (Awareness, USA). 

### Quantification of apoptosis

Apoptosis was assessed through annexin V staining by flow-cytometry in untransfected, mock and ANXA1-transfected PC12 cells treated with MPP+. To do this, approximately 6×10^5^cells were plated in 6-well dishes and treated with MPP+ at 37˚C for 24 hours. Cells were washed with PBS and stained with fluorescein isothiocyanate (FITC)-coupled antiannexin V antibody (Abcam, UK) on ice at 4˚C for 20 minutes. Flow cytometry was carried out with a FACSCalibur flow cytometer (Becton Dickinson, USA). Stained cells were considered apoptotic and 10^4^ events were recorded for each analysis. 

### Assessment of oxidative stress

ROS production was measured with 2´,7´-dichlorodihydrofluorescein diacetate (DCFH-DA) staining for all groups of MPP+ treated PC12 cells with a FACSCalibur flow cytometer (Becton Dickinson, USA). DCFH-DA is a non-fluorescent dye that freely penetrates into the cell and is enzymatically hydrolyzed by intracellular esterases to DCFH. This is then oxidized to the fluorescent DCF by ROS. Briefly, cells were seeded in 6-well plates (6×10^5^cells per well) and were pretreated with 800 μM MPP+ for 24 hours and stained with DCFH-DA (10 μM) for 20 mins in room temperature. For each sample, 10^4^ events were recorded using flow-cytometry. 

### Real-time quantitative polymerase chain reaction analysis

Total RNA was extracted from cells using the Trizol reagent. RNA purity was tested by NanoDrop Spectrophotometer (Biochrom WPA, UK). One µg of total RNA was used in a reverse-transcription reaction utilizing random hexamer primers according to the manufacturer’s protocol (TaKaRa, Japan). Next, 1 μL of the resulting cDNA was amplified in the Step One plus Real-Time PCR thermal cycler (Applied Biosystems, USA) in a total volume of 10 μL containing 5 μL of SYBR Green Master Mix (ROX) (TaKaRa, Japan) along with gene-specific primers. Transcript levels of target genes were normalized to the expression level of *GAPDH*. Differential expression was analyzed according to the 2^-ΔΔCt^ method. Primer pairs (Table 1) were designed by the Beacon designer (Version 7.2, premierbiosoft company, CA, USA) and Perl-primer softwares and synthesized by Metabion (Germany). 

**Table 1:** List of primers


Gene	Primer sequence (5´-3´)	Product length (bp)

ANXA1 (Human)	F:GAGGACTTTGGTGTGAATG	114
	R: GGTGGTAAGGATGGTATTG	
IL-6 (Rat)	F:TCCAGCCAGTTGCCTTCTTG	106
	R: GGTCTGTTGTGGGTGGTATCC	
*iNOS* (Rat)	F:AAGAGACGCACAGGCAGAG	125
	R: CAGGCACACGCAATGATGG	
*GAPDH (Rat)*	F: TGCCGCCTGGAGAAACC	121
	R: TGAAGTCGCAGGAGACAACC	


### Western blot analysis

Protein isolation was performed simultaneously with RNA extraction from the cells. Briefly, 30 µg of extracted protein was loaded on sodium dodecyl sulfate polyacrylamide gel electrophoresis (SDS-PAGE). Proteins were then transferred onto a polyvinylidene fluoride (PVDF) membrane (Biorad, Germany), and blocked with 5% (w/v) non-fat dried milk (Merck, Germany) in PBS. The membrane was labeled with the rabbit anti-NF-κB p65 antibody (1:500, Abcam) or anti-GAPDH (Dako Cytomation, Denmark). The secondary anti body was goat anti-rabbit IgG-HRP (1:16000, Santa Cruz, USA). In case of the Flag antibody, HRPconjugated mouse anti-Flag antibody was used. Immunoreactive bands were visualized using an Amersham enhanced chemiluminescence (ECL) Advance Western Blotting Detection Kit (GE Healthcare, Germany). Western blots were carried out in triplicate from three separate cultures. The band intensity of NF-κB and p65 was compared that of *GAPDH*. 

## Statistical analysis

All experiments were performed at least under three independent treatments of replicated observations. Under each treatment, samples were tested in triplicate. SPSS 18.0 (IBM, IL, USA) was used for statistical analysis and data were expressed as mean ± SEM. Data were analyzed by the independent t test and one-way ANOVA. Furthermore, for all analysis, P<0.05 was considered statistically significant. 

## Results

### PC12 cells overexpressing ANXA1 have normal morphology

**Fig.1:**
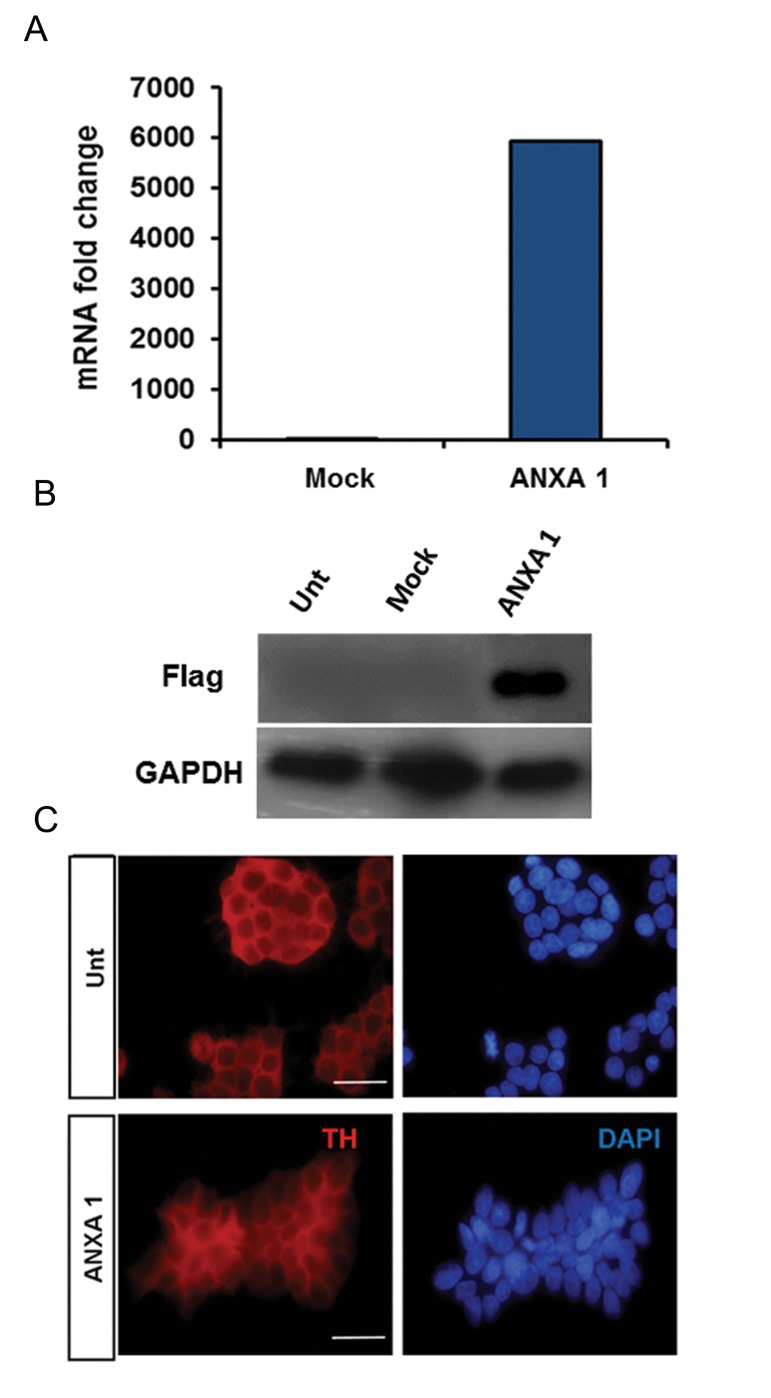
Characterization of *ANXA1-FLAG* transfected PC12 cells. A. Ectopic expression of *ANXA1-FLAG*. Relative expression of *ANXA1-FLAG* was quantified and normalized to the amount of *GAPDH* transcript, B. Western blot analysis to quantify the protein content of *ANXA1-FLAG*. *GAPDH* was used as a loading control, and C. PC12 cells were stained with an antibody against TH. Nuclei were counterstained with 4′, 6-diamidino-2-phenylindole (DAPI). Bar is 200 µm. It should be noted that the staining pattern in ANXA1 transfected cells was similar to untransfected cells (Unt). ANXA1 and Mock represent stably transfected cells with pEPi FGM18F PGL-268/ANXA1-FLAG and pEPi FGM18F PGL268 plasmids respectively. ANXA1; Annexin A1.

Overexpression of *ANXA1-FLAG* CDS was confirmed by RT-qPCR showing significant expression in transfected cells (Fig.1A). Furthermore, western blot analysis confirmed the results obtained by RT-qPCR (Fig.1B). When stained with anti-TH antibody, transformed cells were morphologically similar to normal un-transfected PC12 cells. By having similar expression of TH (Fig.1C). 

### Viability of PC12 cells induced by MPP+

**Fig.2:**
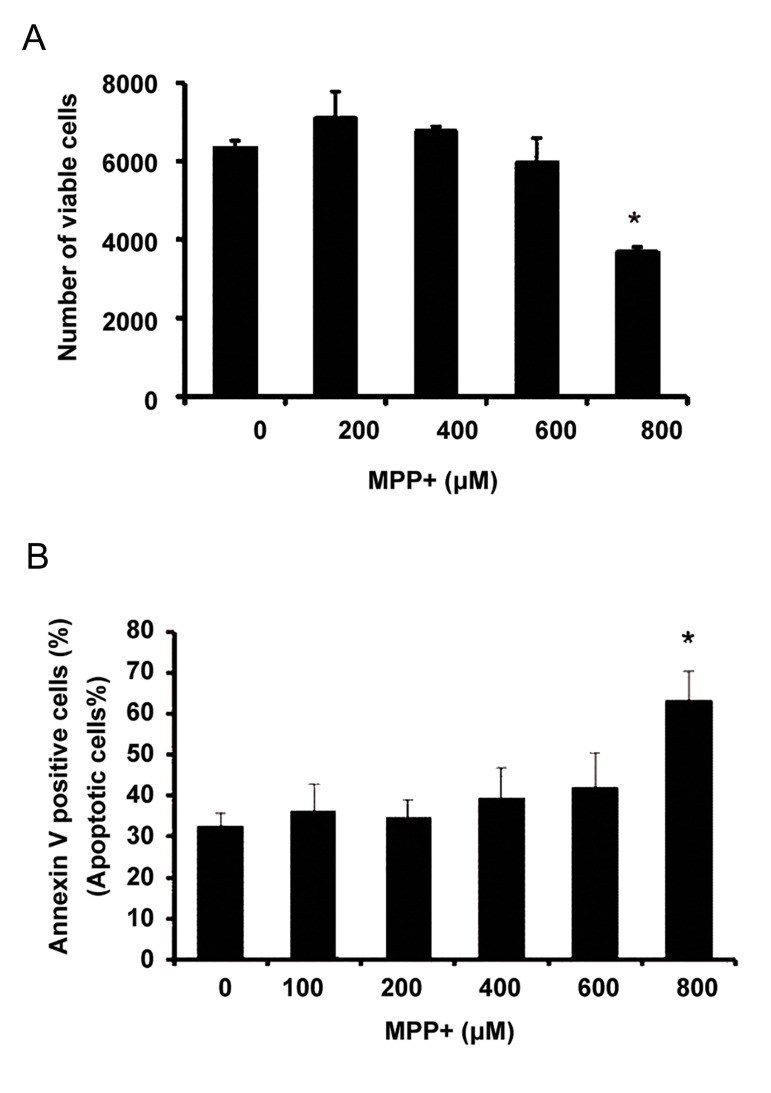
PC12 cell proliferation and apoptosis modulation under MPP+ treatment. A. Viable cell estimation by MTS assay under treatment with different concentrations of MPP+ and B. Flowcytometry analysis of apoptotic cells under treatment with different concentrations of MPP+. Results were expressed as the percentage of cell number after treating with various concentrations of MPP+ relative to the cell number of the control sample. Values represent mean of triplicate independent experiments ± SEM. *; Indicates significant difference between treated and the control sample (0 µM of MPP+) at P<0.05, MPP+; 1-methyl-4-phenylpyridinium and MTS; (3-(4,5-dimethylthiazol-2-yl)-5-(3-carboxymethoxyphenyl)-2-(4-sulfophenyl)-2H-tetrazolium).

Results of MTS assay revealed that 800 µM of MPP+ significantly reduced the number of viable cells (by 50%) (Fig.2A). Also, flow-cytometry indicated an enhanced rate of apoptosis when 800 µM of MPP+ was applied (Fig.2B). Therefore, further experiments were carried out at this concentration to determine whether ANXA1 could counter the inflammation. 

### PC12 cells overexpressing ANXA1 have a similar apoptosis rate

**Fig.3:**
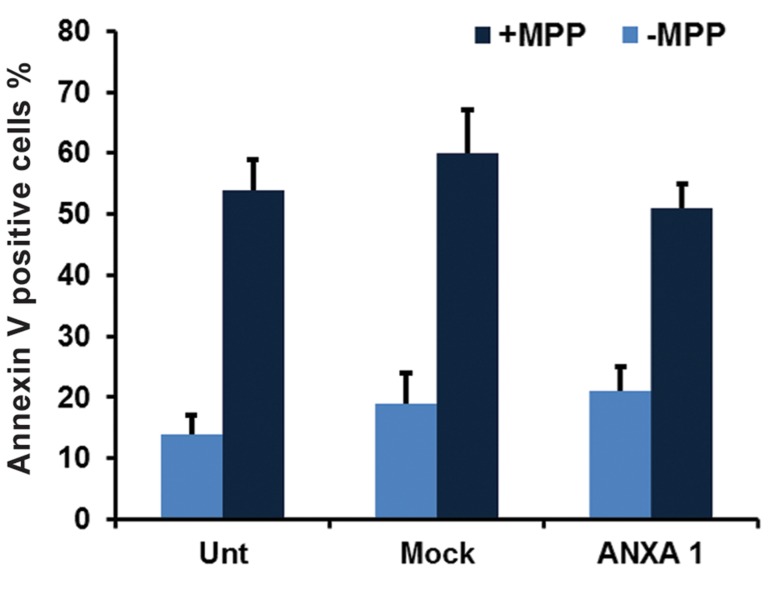
Apoptosis rate constancy in ANXA1 transfected cells. The number of annexin V positive cells was not significantly different between the groups. Results were expressed as the percentage of cell number after treating with various concentrations of MPP+ relative to the cell number of the untreated sample. Values represent mean of triplicate independent experiments ± SEM. ANXA I and Mock represent stably transfected cells with pEPi FGM18F PGL-268/ANXA1-FLAG and pEPi FGM18F PGL-268 plasmids respectively. Unt; Untransformed cells, ANXA1; Annexin A1 and MPP+; 1-methyl-4-phenylpyridinium.

Measurement of apoptosis induced by MPP+ in PC12 cells indicated no significant difference in the number of Annexin V positive cells among groups (Fig.3). This observation indicated that despite the induction of apoptosis by MPP+, *ANXA1-FLAG* was not able to reverse this condition. 

### Overexpression of ANXA1 negatively regulates the reactive oxygen species production and pro-inflammatory factors induced by MPP+ 

**Fig.4:**
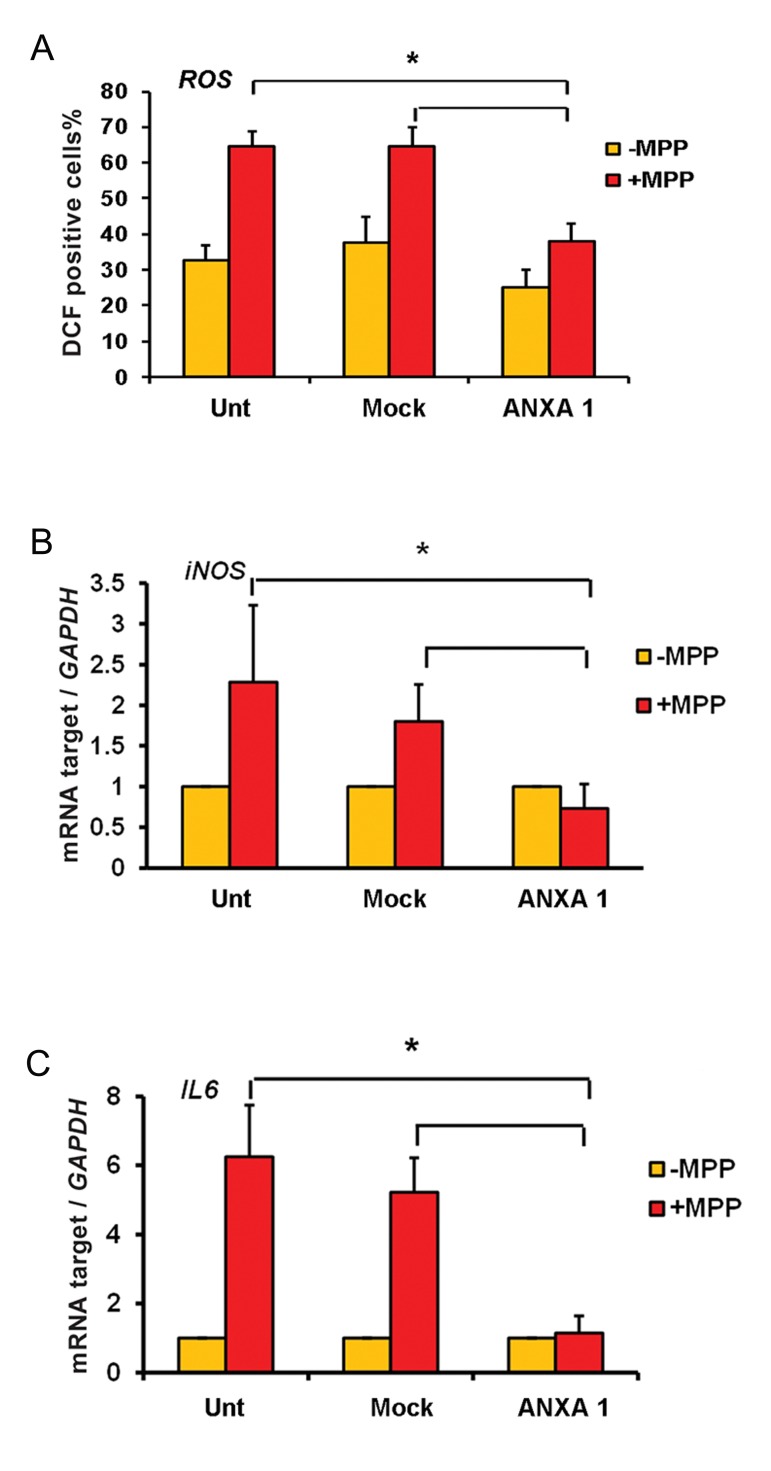
MPP+ induced inflammation was significantly decreased in ANXA1 transformed cells. A. The content of ROS was decreased in ANXA1 transfected cells, B. and C. Relative transcript levels of inflammatory markers, *iNOS* and IL6, were reduced in ANXA1 transfected cells under MPP+ induced inflammatory conditions. *; Indicates significant difference between treated and control samples, Unt; Untransformed cells, ANXA1; Annexin A1, MPP+; 1-methyl-4-phenylpyridinium, *IL-6*; Interlukin-6 and *iNOS*; Inducible nitric oxide synthase.

To evaluate the effect of ANXA1 against intracellular amounts of ROS production, DCFHDA staining was performed. Results showed that positive stained cells (green fluorescence) were significantly more in untransfected and mock groups than the transfected group (P<0.05) (Fig.4A). 

**Fig.5:**
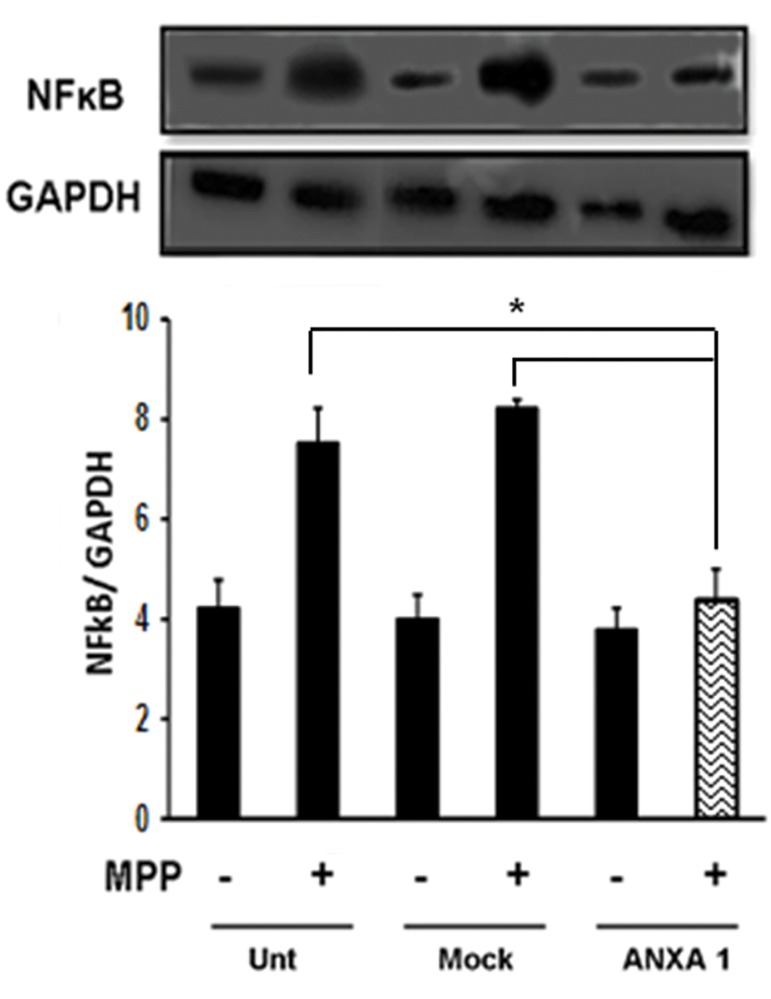
Significant reduction of NF-κB content in ANXA1 transformed cells. *; Indicates significant difference between treated and control samples, ANXA1; Annexin A1 and NF-κB; Nuclear factor-kappa B.

Furthermore, the anti-inflammatory effect of ANXA1 was estimated by two different methods. RT-qPCR results showed transcription levels of *IL-6* and *iNOS* reduced significantly in *ANXA1-FLAG* transfected cells compared with other groups under MPP+ treatment (Fig.4B,C). Western blot results were consistent with those of RT-qPCR. As shown in Figure 5, ANXA1 overexpression was associated with significant reduction of NFκB protein level in transfected cells compared with control groups A B Kiani-Esfahani et al. under MPP+ treatment. These data suggest that *ANXA1-FLAG* expression is capable to decrease transcription levels of *IL-6* and *iNOS* and intracellular amounts of ROS as well as significantly reduce NFκB protein level in PC12 cells. 

## Discussion

In this study, the anti-inflammatory role of ANXA1 was shown. A variety of functions have been attributed to ANXA1 including regulation of cell proliferation, apoptosis, and cell growth (17,18). Although there is no evidence for ANXA1 as a disease-causing gene, it is clear that its expression change can contribute to the pathogenesis of inflammatory diseases and even cancer (28, 29). Our analyses revealed that NF-κB level reduced in ANXA1 transfected cells under inflammation. NF-κB, as a master transcription factor, is activated by various stimuli associated with inflammation. NF-κB activity results in the expression of pro-inflammatory mediators including *IL-1β, IL-6, TNF-α* and *COX-2* (14,16). Our results showed that ANXA1 overexpression also reduced *IL-6* and *iNOS* transcript levels in MPP+ treating transformed PC12 cells. A previous study revealed that ANXA1 expression inhibited transcriptional activity of NF-κB by preventing NF-κB binding to DNA (20). Glucocorticoids, such as dexamethasone, trigger the anti-inflammatory properties through induction of endogenous ANXA1 expression, which is correlated with the inhibition of NF-κB activity (19,30). Our findings revealed that ANXA1 overexpression significantly reduced NFκB content in PC12 cells, suggesting that ANXA1 may play an anti-inflammatory role. Pro-inflammatory cytokines such as *IL-6* are increased in SNpc, striatum, cerebral spinal fluid (CSF) and microglia in PD patients (31,34). These components are the pro-inflammatory mediators released during microglial activation. Although they may initially operate as neuroprotective agents, they act synergistically to produce inflammation-related neuronal damage in later stages (32). Hence, reduced amounts of *IL-6* and other inflammatory parameters under MPP+ treatment in ANXA1 transfected PC12 cells group suggests that ANXA1 plays a prominent role in inflammation recovery and may be applied as a neuroprotective agent when inflammation occurs. 

Oxidative stress is a mediator for nerve cell death in inflammation. Previous studies have reported that apoptotic death of dopaminergic neurons may be initiated by oxidative stress. MPP+ as an active toxin produces oxidative stress in PC12 cells. MPP+ is actively transported to the mitochondria and prevents the function of complex I of the electron transport chain, thus resulting in production of ROS (35). We found that ROS generation and *iNOS* expression were down-regulated in ANXA1 transfected PC12 cells under MPP+ treatment while apoptosis remained unchanged. This suggests that ANXA1 may protect PC12 cells against ROS-induced injuries. A possible explanation for this dichotomy is that apoptosis induction with MPP+ treatment in PC12 cell may have occurred via different pathways in which ANXA1 was not involved. Presumably, MPP+ could stimulate cell death through different mechanisms beside ROS production. Examples of such mechanisms are ER stress, p53 activation, release of cytochrome c from mitochondria, mitochondrial swelling, Ca^2+^ release, p38 activation and other MAP kinase pathways. It remains unknown, however, which of these mechanisms is involved in cell death in MPP+-treated PC12 cells. Further to our findings, Solito et al. (36) reported that exogenous ANXA1 stimulated apoptosis in human neutrophils and endogenous ANXA1 was released from apoptotic neutrophils which stimulated macrophages to phagocyte these apoptotic cells (37). LPS-induced endotoxic shock in ANXA1–/– mice was also correlated with increase in TNFα, IL-1 and *IL-6* levels in the blood when compared with wild-type mice (19). Taken together, ANXA1 is unlikely to be involved in inhibition of apoptosis in PC12 cells as a dopaminergic cells. ANXA1 may act as a downregulator for the innate immune cells, in particular in generation of pro-inflammatory mediators. Interestingly, exogenous ANXA1 or an N-terminal portion of ANXA1 peptide, was able to ameliorate inhibitory effects of glucocorticoids, including inhibition of leukocyte recruitment at inflammatory sites (38). 

## Conclusion

ANXA1, as a potent neuroprotective agent, may prevent mitochondrial functions from damage and interfere with the generation of ROS, *iNOS* and pro-inflammatory mediators. One of the future directions of this study is to extend these experiments to differentiated PC12 cells or primary cultured dopaminergic cells. 
